# The longitudinal effect of subclinical hypothyroidism on urine microalbumin-to-urine creatinine ratio in patients with type 2 diabetes mellitus

**DOI:** 10.1186/s12902-019-0405-0

**Published:** 2019-08-05

**Authors:** Juan Xie, Xiaoqing Wang, Yiyuan Zhang, Hailun Li, Yong Xu, Donghui Zheng

**Affiliations:** The Affiliated Huai’an Hospital of Xuzhou Medical University, Huaian, 223002 Jiangsu China

**Keywords:** Subclinical hypothyroidism, Type 2 diabetes mellitus, Urine microalbumin-to-urine creatinine ratio

## Abstract

**Background:**

In patients with diabetes mellitus, the urinary microalbumin-to-urine creatinine ratio (UACR) can not only predict the occurrence of diabetic nephropathy but also can be a risk factor for cardiovascular disease and renal function damage. Current studies on subclinical hypothyroidism (SCH) and UACR are mainly cross-sectional studies, and the results suggest that SCH is an independent risk factor for UACR. To further explore the longitudinal effect of SCH on UACR, we carried out this study.

**Methods:**

This was a retrospective cohort study including 46 patients with type 2 diabetes mellitus and SCH in the Department of Endocrinology, The Affiliated Huai’an Hospital of Xuzhou Medical University from January 2013 to April 2018. At the same time, 96 patients with type 2 diabetes mellitus and euthyroid were chosen according to 1:2 approximately matched with age, sex and duration of diabetes mellitus. Univariate analysis, stratified analysis, and multiple linear regression analysis were used to investigate the effect of SCH on ΔUACR(ΔUACR = UACR after 1 year - baseline UACR) in patients with type 2 diabetes mellitus.

**Results:**

There was no significant difference between the baseline UACR, (*p* > 0.05). However, the ΔUACR was significantly higher in SCH group than euthyroid group, as shown by univariate analysis, stratified analysis and multiple linear regression analysis (β:-1.071, 95% CI: − 1.713--0.428), and the difference was statistically significant (all *p* < 0.05).

**Conclusion:**

SCH is associated with an increased UACR in type 2 diabetes mellitus patients. It is necessary to screen for thyroid function in type 2 diabetes mellitus and increase the follow-up frequency of UACR in patients with SCH.

## Background

Subclinical hypothyroidism (SCH) is an endocrine disorder characterized by elevated serum thyroid stimulating hormone (TSH) levels and normal thyroid hormone (T4) and triiodothyronine (T3) concentrations. The prevalence of SCH is approximately 5% in the general population [1–3], but is higher in type 2 diabetes mellitus (T2DM), the reported SCH levels in DM is roughly 10% [4–6]. Higher morbidity is related to age, being female and iodine intake [7]. SCH is an early mild hypothyroidism that can develop into clinical hypothyroidism, which is a risk factor for atherosclerosis, heart failure, atrial fibrillation, stroke, and renal dysfunction [8, 9].

Urinary microalbumin-to-urine creatinine ratio (UACR) is an important predictor of diabetic nephropathy, and independent risk factor for cardiovascular disease and represents endothelial dysfunction in DM, even within the normal range [10, 11]. A number of cross-sectional clinical investigations suggest that patients with DM and SCH have higher urinary microalbumin levels than those with normal thyroid function [12–14], high TSH was significantly associated with microalbuminuria even in diabetic patients with euthyroid [15]. The longitudinal effect of SCH on UACR in patients with type 2 diabetes mellitus is still unclear. We retrospectively analyzed the effect of SCH on the ΔUACR (ΔUACR = UACR after 1 year - baseline UACR) in patients with T2DM to provide a theoretical basis for the follow-up and treatment of SCH in T2DM, which may delay the occurrence and progression of diabetic nephropathy and improve the long-term prognosis of patients.

## Methods

### Patients

This is a retrospective cohort study using data from Electronic Medical Record System of The Affiliated Huai’an Hospital of Xuzhou Medical University. From January 2013 to April 2018, 46 patients with T2DM and SCH were recruited. At the same time, 96 patients with T2DM and euthyroid were chosen according to 1:2 approximately, who were matched with regards to age, sex and duration of DM. Subjects with age ≥ 18 years old, a diagnosis of T2DM, and have complete clinical data were included. The exclusion criteria were (1) history of thyroid diseases, or taking levothyroxine tablets (2) stage 4–5 chronic renal failure, (3) glomerulonephritis, (4) liver dysfunction, (5) severe infection, (6) connective tissue disease, (7) malignant tumor, (8) pregnancy, (9) women taking contraceptives or hormone replacement therapy, (10) uncontrolled hypertension, (11)congestive heart failure and (12) amiodarone or lithium preparations. The diagnosis of DM was based on the criteria of the 1999 WHO criteria, and the diagnosis of SCH was persistently elevated TSH values (at least twice, at least 3 months apart) with FT4 levels within the reference range, exclusion of previous thyroid disease.

### Data collection

Patient baseline data were obtained from Electronic Medical Record System, included age, gender, height, body weight, blood pressure, history of hypertension, duration of T2DM, total cholesterol (TC), triglycerides (TG), high-density lipoprotein cholesterol (HDL-C), low-density lipoprotein cholesterol (LDL-C), serum creatinine, blood urea nitrogen, glycosylated hemoglobin (HbA1c), FT4, FT3, TSH, and UACR. After 1 year, the patients were readmitted to the hospital, and we collected all the informations above.

### Laboratory measurements

Blood specimens were tested in the central laboratory and urine specimens in the nephropathy laboratory. UACR was measured from a single voided urine sample by Nephropathy Laboratory. Detection of TSH, FT3 and FT4 by chemiluminescent particle immunoassay (Abbot, America, i2000SR). The reference ranges of thyroid function test in our hospital: TSH 0.35–5.0 mU/ml; FT3 2.75–6.8 pmol/L, FT4 7.5–22 pmol/L. Urinary creatinine was detected by enzymatic method(SIEMENS, Germany, BNProSpec); urinary albumin was detected by scattering turbidimetry(Abbot, America,c16000).

### Related definitions

Glycemic control: According to the guidelines for good glycemic control, the glycosylated hemoglobin (HbA1c) level should be controlled at a level of < 7% for most patients with T2DM, poor control of glycemic was considered when HbA1c > 7% [16, 17]. BMI: BMI < 23.9 was defined normal, BMI > 24.0 was defined overweight or obesity.

### Medical formulas

UACR (mg/mmol) = urinary albumin (mg/L)/ urinary creatinine (mmol/L). ΔUACR = UACR after 1 year - the baseline UACR. Body mass index (BMI) was derived as weight (kg)/height (m)^2^. Estimated glomerular filtration rate (eGFR) [mL/(min·1.73 m^2^)] = 186 × Scr(mg/dL)^-1.154^ × age^− 0.203^(× 0.742 female).

### Statistical analysis

All statistical tests were analyzed by SPSS version 23.0. The quantitative normal distribution data were expressed as the means±standard deviation (SD), and the classification data were described in percentage form. The comparison of continuous data was achieved by *t-*test or rank sum test, and the classification data were compared with *X*^2^ test. Univariate analysis, stratified analysis and multiple linear regression analysis were used to explore the effect of SCH on ΔUACR. A two-sided *p*-value <0.05 was considered to be statistically significant.

## Results

The baseline characteristics of study patients are presented in Table 1. Patients with SCH had higher creatinine, lower e-GFR, higher TSH, lower TG and FT3 than euthyroid patients(all *P* < 0.05). There were no significant differences in gender, age, duration of DM, history of hypertension, BMI, FT4, TC, HDL-C and LDL-C, baseline UACR between the two groups (all *P* > 0.05).

**Table 1:** Baseline characteristics of study participants

Characteristic	SCH (*n* = 46)	Euthyroid (*n* = 96)	*P*-value
Gender (male/female)^b^	23/23	49/47	0.91
Duration of T2DM (years)^a^	12.74 ± 10.40	11.63 ± 6.79	0.45
History of hypertension (have/none)^b^	15/31	25/71	0.42
BMI (kg/m^2^) ^a^	25.14 ± 2.86	25.21 ± 2.98	0.89
TC (mmol/L)^a^	2.08 ± 1.15	2.13 ± 2.69	0.90
TG (mmol/L)^a^	4.20 ± 0.98	4.37 ± 1.56	0.51
HDL-C (mmol/L)^a^	1.09 ± 0.30	1.17 ± 0.29	0.10
LDL-C (mmol/L)^a^	2.18 ± 0.66	2.29 ± 0.72	0.38
Baseline creatinine (μmol/L)^b^	77.80 ± 27.19	67.25 ± 17.66	0.01
Baseline eGFR (ml/min)^b^	90.49 ± 35.04	103.03 ± 32.26	0.04
Baseline UACR (mg/mmol)^b^	4.82 ± 8.38	3.68 ± 6.25	0.64
TSH (μIU/ml) ^b^	8.57 ± 6.31	2.09 ± 0.96	0.00
FT3 (mU/ml)^b^	4.12 ± 0.88	4.42 ± 0.70	0.03
FT4 (mU/ml)^b^	17.61 ± 14.66	16.71 ± 2.49	0.68

^a^, Expressed as the means ± standard deviation (SD), the comparison of continuous data was achieved by *t-*test or rank sum test; ^b^, Described in percentage form. The classification data were compared with *X*^2^ test. *SCH* subclinical hypothyroidism,*T2DM* type 2 diabetes mellitus, *BMI* body mass index, *eGFR* estimated glomerular filtration rate, *UACR* urinary microalbumin-to-urine creatinine ratio, *TC* total cholesterol, *TG* triglyceride, *HDL-C* high-density lipoprotein cholesterol, *LDL-C* low-density lipoprotein cholesterol, *TSH* thyroid stimulating hormone, *FT4* free thyroid hormone, *FT3* triiodothyronine

The longitudinal effect of SCH on UACR in patients with type 2 diabetes mellitus: 1. Univariate analysis: The ΔUACR in the SCH group was higher than in the euthyroid group, and the difference was statistically significant (*P* < 0.001), (Table 2). 2.Stratified analysis: When stratified according to sex (male/female), duration of diabetes (duration≤10 years/duration> 10 years), BMI (BMI < 24 kg/m^2^/BMI>24 kg/m^2^), control of glycemic (well/poor), history of hypertension (have/none), the ΔUACR of the SCH group was higher than euthyroid group. The difference was statistically significant (*p* < 0.01), (Table 3). 3. Multivariate analysis: After converting the absolute value of ΔUACR to logarithm, it obeyed normal distribution. The BMI (1 was normal; 2 as overweight and obesity), control of glycemic (1 was well controlled; 2 was poorly controlled), the duration of diabetes mellitus (1 for 10 years or less; 2 for more than 10 years), age (1 for 18 to 65 years; 2 for 66 to 75 years, 3 for more than 75 years) were controlled, using multiple linear regression analysis of the effect of SCH on ΔUACR. The results showed that ΔUACR in the SCH group was still statistically significant compared with that in the control group (*p* < 0.01) (Table 4).

**Table 2:** Univariate analysis for ΔUACR in patients with T2DM

Group	Median	P25-P75	*P*-value
SCH	1.07	(− 0.02,6.34)	< 0.001
Euthyroid	0.00	(− 0.68,0.60)	

Analysis is achieved by rank sum test

**Table 3:** Stratified analysis for ΔUACR in patients with T2DM

stratified factors	Euthyroid M (Q)	SCH M(Q)	*P*-value^&^
Gender			
male	0.10 (−0.40,1.20)	1.20 (0.00,6.62)	< 0.01
female	-0.20 (− 0.74,0.30)	0.80 (− 0.10,6.24)	< 0.01
BMI(kg/m^2^)			
normal	0.25 (−0.49,1.75)	1.140 (0.40,16.75)	< 0.01
overweight or obesity	−0.10(− 0.73,0.27)	1.00 (− 0.09, 5.96)	< 0.01
Duration of T2DM (years)			
~ 10	−0.10 (− 0.71,0.38)	1.17 (− 0.03,12.13)	< 0.01
11~	0.04 (− 0.56,0.93)	0.75 (− 0.02,6.34)	< 0.01
Control of glycemic			
well	0.00 (−0.60,0.60)	1.40 (0.18,5.68)	< 0.01
poor	0.00 (−0.73,0.65)	0.84 (−0.08,7.47)	< 0.01
History of hypertension			
have	−0.10 (− 0.75,1.23)	0.90 (0.00,4.60)	< 0.01
none	0.00 (−0.50,0.39)	1.20 (−0.07,10.00)	< 0.01

*M* median, *Q* interquartile range (P25,P75); &, Analysis is achieved by rank sum test, *BMI* body mass index; HbAlc < 7% was better glycemic controlled; HbAlc > 7% was poor glycemic controlled; BMI < 23.9 was defined normal; BMI > 24.0 was defined overweight or obesity

**Table 4:** Multivariate analysis for ΔUACR in patients with T2DM

	β	SD	*P*-value	95% CI	
Group	−1.07	0.33	0.00	−1.71	− 0.43
BMI	−0.66	0.33	0.05	−1.32	−0.00
Control of glycemic	0.19	0.331	0.561	−0.46	0.85
Duration of T2DM	−0.25	0.30	0.42	−0.85	0.35
Age	−0.05	0.19	0.78	−0.44	0.33
Constant term	2.80	1.14	0.02	0.54	5.07

After converting the absolute value of ΔUACR to logarithm, it obeyed normal distribution, the absolute value of UACR was used to analysis. *SD* standard deviation, *T2DM* type 2 diabetes mellitus, *BMI* body mass index

## Discussion

Thyroid dysfunction and T2DM are the two most common diseases observed in the Department of Endocrinology. Studies have shown that the prevalence of thyroid diseases is significantly higher in patients with DM than healthy people, and SCH at the first list [7, 18]. SCH can be seen as mild thyroid failure [19], and although most SCH patients have no obvious symptoms, they are more likely to suffer from depression, fatigue, muscle weakness, cold tolerance, and quality of life, cognitive function and memory decline than healthy people [20]. Several studies showed that subclinical hypothyroidism is associated with dyslipidemia, hypertension, accelerated atherosclerosis, and coronary artery disease, heart failure, lower renal function [21–24].

Yasuda T.et al. reported that SCH is independently associated with albuminuria in patients with T2DM on the cross section [12]. Subsequently several studies have come to the same conclusion [25, 26], which suggests that subclinical hypothyroidism may have an impact on urinary microalbumin. In this study, the ΔUACR in the SCH group was statistically significant, whether in univariate analysis, stratified analysis or multivariate analysis. Our study further suggests that SCH has an effect on UACR in patients with T2DM and promotes the progress of UACR. As far as we know, this is the first study about the effect of SCH on the 1-year change UACR in patients with T2DM.

The mechanism of SCH affecting UACR in patients with T2DM is unclear. The possible mechanisms are as follows. Hypothyroidism and SCH are related to insulin sensitivity and impaired glucose tolerance, and the ability of insulin to utilize glucose in muscle decreases [27]. The downregulation of glucose transporter protein directly affects the degradation of insulin [28]. Second, some studies suggest that endothelial dysfunction occurs in patients with SCH due to endothelium-dependent vasodilation dysfunction and NO utilization disorder, and this phenomenon still independently appears after correcting lipid disorders and levothyroxine treatment [29].

In this study, the sample size of patients was small. The control group was matched for age, gender, and duration of DM, so there may be case selection bias. To reduce selection bias, the hospitalization number of patients with T2DM with euthyroid was randomized, and 96 patients were selected as a control group using a randomization tool after calculating the sample size of the control group. The specific mechanism of subclinical hypothyroidism affecting UACR in patients with T2DM is still unclear, and further basic research is needed.

## Conclusions

SCH has a significant impact on UACR in patients with T2DM. It is necessary to screen for thyroid function in patients with T2DM. At the same time, we need to increase the frequency of follow-up of UACR in patients with T2DM and SCH, with timely intervention if necessary, to improve the prognosis of patients.

## Data Availability

Patients’ information are not publicly available due to restrictions imposed by China law, is may be obtained from hospital electronic medical record system on reasonable request and if legal implications are fulfilled. The names of patients were not registered in our data and their unique ID numbers were locked for confidentiality.

## Abbreviations

ADA: American Diabetes Association
BMI: Body mass index
eGFR: Estimated glomerular filtration rate
FT3: Triiodothyronine
FT4: Free thyroid hormone
HbA1c: Glycosylated hemoglobin
HDL-C: High-density lipoprotein cholesterol
LDL-C: Low-density lipoprotein cholesterol
SCH: Subclinical hypothyroidism
SD: Standard deviation
T2DM: Type 2 diabetes mellitus
TC: Total cholesterol
TG: Triglyceride
TSH: Thyroid stimulating hormone
UACR: Urinary microalbumin-to-urine creatinine ratio
